# Investigations of the relationship betw een disease and airborne (1→3)-β-D-glucan in buildings

**DOI:** 10.1080/09629359791613

**Published:** 1997-11

**Authors:** Ragnar Rylander

**Affiliations:** 1 Department of Environmental Medicine University of Gothenburg Medicinaregatan 16 Gothenburg S-413 90 Sweden

## Abstract

Studies on the relationship between symptoms in indoor air and the amount of airborne (1→3)-β-D-glucan were reviewed. Relationships were found for symptoms and objective tests of airways inflammation. The data suggest that (1→3)-β-D-glucan could be a causative agent.


Mediators of Inflammation, **6,** 275–277 (1997)

### Investigations of the relationship between disease and airborne (1→3)-β-D-glucan in buildings

**Ragnar Rylander**

Department of Environmental Medicine, University of Gothenburg, Medicinaregatan 16, S-413 90 Gothenburg, Sweden

Tel: +46 31 7733601
Fax: +46 31 825004
Email: ragnar.rylander@envmed.gu.se

**Studies on the relationship between symptoms in indoor air and the amount of airborne (1→3)-β-D-glucan were reviewed. Relationships were found for symptoms and objective tests of airways inflammation. The data suggest that (1→3)-β-D-glucan could be a causative agent.**

**Keywords:** Indoor air, airways inflammation

### Introduction

The presence of extended problems in relation to indoor environments has been recognized in a large number of studies from different countries [1,2]. Commonly reported symptoms are irritation in the eyes, nose and throat, dry cough, as well as fatigue/headache and skin problems. The consistency in the symptom profile is remarkable, suggesting that most cases may have a common causative agent.

Studies during recent years suggest that the symptoms reflect a non-specific airways inflammation [3]. A specific, classical immunoglobulin E-mediated sensitization can develop against an indoor air antigen such as house dust mite or molds, but this occurs only in a small proportion of exposed populations. In that respect, symptoms among persons in indoor air environments are very similar to those present among persons working in organic dust environments such as swine confinement buildings, poultry houses and composting facilities [4].

A large number of studies show a relationship between airways inflammation and a history of dampness or flooding of buildings. Humidity in building structures favors the growth of fungi and certain bacteria, generally referred to as molds. These microorganisms contain several agents with important biological properties such as endotoxin, mycotoxins and (1→3)-β-D-glucan.

(1→3)-β-D-glucan is a polyglucose compound in which the sugar rings are joined in a β-1→3-position. This steric form is responsible for the effects of (1→3)-β-D-glucan on the immune system of the body. There is an abundant literature showing that (1→3)-β-D-glucan interferes with macrophage function, activates lymphocyte function and works synergistically with other agents to cause inflammation [5–8].

In view of the important biological potency of (1→3)-β-D-glucan, our laboratory has undertaken a series of field investigations to evaluate the relationship between (1→3)-β-D-glucan in indoor air and the presence of airways inflammation. These studies are reviewed here.

### Measurements of (1→3)-β-D-glucan

### Sampling of airborne particles

Previous experience has demonstrated the need to generate air movements to suspend floor dust to obtain relevant exposure data for airborne dust indoors [9]. In order to generate a dust aerosol, a device producing an airstream directed at the floor was passed over the floor for 5 min at the beginning of each of two 15-min measuring periods.

For the aerosol sampling, air was drawn through a filter (ATTD, 0.8 μm Millipore, Cambridge, Massachusetts, USA) with a volume of about 2 liters/min for 30 min. The filters were situated 1.2 m above the ground.

### Analyses

The analysis of (1→3)-β-D-glucan was performed according to previously described techniques [10]. The filters were shaken on ice for 10 min at room temperature in 0.3 N NaOH. This process unwinds

© 1997 Rapid Science Publishers 

the triple helix of the (1→3)-β-D-glucan and renders it water-soluble for a limited time. A 50-μl portion of the samples was placed in hollows of a microtiter plate together with a glucan-specific Limulus lysate, containing the azodye required for the color test (Fungitec G test; Seikagaku Corporation, Tokyo, Japan). The sample was incubated in a Wellreader (Seikagaku Corporation) and the kinetics of the color reaction was read photometrically and transformed into absorbance units at the maximum slope of the curve. This value was compared to a standard of a water-soluble (1→3)-β-D-glucan provided by the manufacturers (Pachyman–Seikagaku Corporation, Tokyo, Japan) and the results were expressed as units of (1→3)-β-D-glucan per milliliter liquid. The value for air flow through the filter was then used to transform this value to $ng/m^3$.

### Studies performed

The first study was an investigation of a number of flats in a small town in Sweden [11]. The presence of symptoms was evaluated by questionnaire (35 respondents) and measurements of (1→3)-β-D-glucan were made in 22 flats. Due to differences in the analysis method, the values found are not directly comparable to levels reported using the current analytical method (see above) but an extrapolation demonstrates that the values ranged up to 20 $ng/m^3$. For this reason, the exposure was divided into three classes: high-, medium- and low-glucan. The results showed that the higher the class of (1→3)-β-D-glucan, the larger the proportion of respondents who reported symptoms indicative of airways inflammation (Table 1). The same was found for tiredness and headache.

**Table 1.** Airborne classes of (1→3)-β-D-glucan in relation to extent of symptoms (percentage of subjects expressing symptoms)

| Glucan class | Low | Medium | High |
|---|---|---|---|
| *n* | 10 | 17 | 8 |
| Nasal irritation | 20 | 29 | 38 |
| Throat irritation | 20 | 29 | 50 |
| Dry cough | 20 | 12 | 38 |
| Headache | 2 | 5 | 30 |
| Tiredness | 21 | 58 | 75 |

The second study involved a number of buildings for which complaints of indoor air-related symptoms had been made: a daycare center, a post office and two primary schools [9]. An office building where no symptoms had been reported served as a control. A total of 39 persons in the buildings with symptoms and 405 persons in the control building were investigated, and measurements were made of the amount of airborne endotoxin and (1→3)-β-D-glucan. The results are summarized in Table 2.

Airborne levels of (1→3)-β-D-glucan ranged from 0.06 to 0.55 $ng/m^3$ or approximately up to 6 $ng/m^3$ with the current method of analysis. The extent of different symptoms was related to levels of airborne (1→3)-β-D-glucan.

**Table 2.** Airborne levels of (1→3)-β-D-glucan in relation to extent of symptoms (percentage of subjects expressing symptoms)

| | Control | Daycare/post office | School |
|---|---|---|---|
| *n* | 405 | 19 | 20 |
| Glucan ($ng/m^3$) | <0.1 | 1.3 | 5.2 |
| Nasal irritation (%) | 16 | 37 | 50 |
| Throat irritation (%) | 11 | 37 | 45 |
| Dry cough (%) | 6 | 5 | 35 |
| Headache (%) | 2 | 5 | 30 |
| Tiredness (%) | 21 | 58 | 75 |

A case study in Switzerland comprised a clinical investigation of two boys living in a house displaying indoor mold and with airborne (1→3)-β-D-glucan levels ranging between 5 and 106 $ng/m^3$ (mean 41.9) [12]. The boys developed an airways inflammation with coughing, wheezing and tiredness after about 6 months of living in the house and one of them became sensitized to house-dust mite. They moved out of the house and the symptoms disappeared. About a year later, the parents developed airways inflammation and they also had to move out of the house.

Another study was performed in a daycare center with a history of dampness [13]. The cement slab on which the building was erected was placed directly on the ground with inadequate drainage arrangements. For several years, symptoms of nasal swelling, throat irritation, headache and tiredness had been reported by the staff at the center. One part of the study was made when the symptoms were present (May 1992). A few months later, the center was closed for renovation and the personnel worked elsewhere. After renovation, they returned to the center and were re-examined in April 1994 and June 1995.

In this study, airway responsiveness was measured using a methacholine challenge test. At the second re-examination 2 years afterwards, there were 11 subjects who had been present at all examinations. These comprised the study group.

Before the renovation, the measurment of airborne (1→3)-β-D-glucan was 11.4 $ng/m^3$ ($n = 24$, SD 2.3). After the renovation the value was 1.3 $ng/m^3$ ($n = 13$, SD 0.9), which was significantly lower ($P < 0.0001$). Measurements of airway responsiveness are shown in Table 3. The average decrease in forced expiratory volume in 1 s ($FEV_1$) after methacholine challenge was lower 2 and 3 years after the renovation than be-

**Table 3.** Airway responsiveness before and after building renovation, expressed as change in forced expiratory volume in 1 s ($FEV_1$) after inhalation of 1.2 mg methacholine

| | Before | 2 years | 3 years |
|---|---|---|---|
| $FEV_1$ | –7.8 | –4.0 | –5.9 |
| *n* >4% | 8 | 2 | 3 |

*n*, number of subjects showing a decrease in $FEV_1$ of over 4%.

fore ($P = 0.006$ for permutation test before versus 2 years, NS before versus 3 years). As shown, the number of persons with a decrease in $FEV_1$ larger than 4% was lower at 2 and 3 years ($P = 0.03$ and 0.08, $\chi^2$, Yate's correction).

Over the years, a number of measurements have been performed in private homes and some other locations, initiated by reports of symptoms of airways inflammation. Other measurements have been made in indoor environments where no symptoms have been reported. These measurements cannot be looked upon as a representative sample of indoor environments, but the data nevertheless contain information of value in determining the effects of (1→3)-β-D-glucan. Measurements have been performed in 20 houses with symptoms and 13 without symptoms. In cases where several measurements were made in one location, such as bedrooms, kitchen and sitting room, a mean value was calculated on the rationale that persons in a dwelling move between different rooms.

The results (Table 4) showed that the average level of (1→3)-β-D-glucan in houses inhabited by people reporting symptoms was significantly higher than in houses inhabited by people reporting no symptoms ($P < 0.0019$). A suggested cut-off point is around 5 ng/m$^3$, and only a few houses inhabited by people without symptoms exceeded that value. A level of 10 ng/m$^3$ and above was always related to symptoms.

**Table 4.** Airborne levels of (1→3)-β-D-glucan in buildings inhabited by people with and without symptoms

| | Symptoms | No symptoms |
|---|---|---|
| *n* | 20 | 13 |
| Mean level (ng/m$^3$) | 8.6 | 2.5 |
| Range (ng/m$^3$) | 1.9–32 | 0.4–5.7 |
| *n* >5 ng/m$^3$ (%) | 16 (80) | 2 (15) |
| *n* >6 ng/m$^3$ (%) | 12 (60) | 0 (0) |

*n*, number of houses.

### Discussion

There are considerable methodological problems in sampling room dust. To obtain sufficient amounts, vacuum-cleaning of surfaces or materials such as mattresses is often used [14,15]. This technique also samples particle sizes which are not respirable, and in the studies reported here the sampling was focused on particles which could deposit at all levels of the respiratory tree. The technique chosen was based on previous work [11] showing that a certain amount of activity in the room was required to obtain measurable amounts of airborne particles as well as (1→3)-β-D-glucan.

A general conclusion from the studies reviewed here is that there was a relationship between exposure to (1→3)-β-D-glucan and the extent of symptoms of airways inflammation. There was also a relationship to the general extent of symptoms of fatigue and headache. The causative factor for these symptoms may be a general distribution of inflammatory mediators from activated cells in the lung [16]. In some of the studies, dose–response relationships were demonstrated. However, a final conclusion regarding causality requires experimental challenges to the pure substance.

### Acknowledgements

Our own work reported here was supported by the Center for Indoor Air Research (contract 90-25). The specific lysate was provided by Dr S. Tanaka, Seikagaku Kogyo Corporation, Tokyo, Japan.

### References

1. Burge PS, Hedge A, Wilson S, Bass JH, Robertson A: Sick buildings syndrome: a study of 4373 office workers. *Ann Occup Hyg* 1987, **31**:493–504.
2. Samet JM, Spengler JD (eds): *Indoor Air Pollution: A Health Perspective*. Baltimore: Johns Hopkins University Press; 1991:1–408.
3. Rylander R. Sick building syndrome. In: *XVI European Congress of Allergology and Clinical Immunology*. Edited by Basomba A, Sastre J. Madrid: Monduzzi Editore; 1995:409–414.
4. Rylander R, Jacobs RR (eds): *Organic Dusts: Exposure, Effects, and Prevention*. Boca Raton: CRC Press; 1994.
5. Di Luzio NR: Update on the immunomodulating activities of glucans. *Springer Semin Immunopathol* 1985, **8**:387–400.
6. Fogelmark B, Sjöstrand M, Rylander R: Pulmonary inflammation induced by repeated inhalations of (1→3)-β-D-glucan and endotoxin. *Int J Exp Pathol* 1994, **75**:85–90.
7. Ohno N, Miura NN, Chiba N, Adachi Y, Yadomae T: Comparison of the immunopharmacological activities of triple and single-helical Schizophyllan in mice. *Biol Pharm Bull* 1995, **18**:1242–1247.
8. Williams D: Overview of (1→3)-β-D-glucan chemistry, immunobiology and toxicology. In: *Proceedings of the 20th Cotton and Other Organic Dust Conference*. Nashville: National Cotton Council; 1996:285–289.
9. Rylander R, Persson K, Goto H, Yuasa K, Tanaka S: Airborne β,1→3 glucan may be related to symptoms in sick buildings. *Indoor Environ* 1992, **1**:263–267.
10. Tamura H, Arimoto Y, Tanaka S, Yoshida S, Obayashi T, Kawai T: Automated kinetic assay for endotoxin and (1→3)-β-D-glucan in human blood. *Clin Chim Acta* 1994, **226**:109–112.
11. Rylander R, Sörensen S, Goto H, Yuasa K, Tanaka S: The importance of endotoxin and glucan for symptoms in sick buildings. In: *Present and Future of Indoor Air Quality*. Edited by Bieva CJ, Courtois Y, Govaerts M. Amsterdam: Excerpta Medica; 1989:219–226.
12. Rylander R, Hsieh V, Courteheuse C: The first case of sick building syndrome in Switzerland. *Indoor Environ* 1994, **3**:159–162.
13. Rylander R: Airborne (1→3)-β-D-glucan and airway effects in a day care center before and after renovation. *Arch Environ Health* 1997, **52**:281–285.
14. Michel O, Genion R, Duchateau J, Vertongen F, Le Bon B, Sergysels R: Domestic endotoxin exposure and clinical severity of asthma. *Clin Exp Allergy* 1991, **21**:441–448.
15. Michel O, Genion R, Le Bon B, Content J, Sergysels R, Duchateau J: Inflammatory response to acute inhalation of endotoxin in asthmatic patients. *Am Rev Resp Dis* 1992, **146**:352–357.
16. Michel O, Duchateau J, Plat G, *et al.*: Blood inflammatory response to inhaled endotoxin in normal subjects. *Clin Exp Allergy* 1995, **25**:73–79.
